# Decreased epigenetic age of PBMCs from Italian semi-supercentenarians and their offspring

**DOI:** 10.18632/aging.100861

**Published:** 2015-12-15

**Authors:** Steve Horvath, Chiara Pirazzini, Maria Giulia Bacalini, Davide Gentilini, Anna Maria Di Blasio, Massimo Delledonne, Daniela Mari, Beatrice Arosio, Daniela Monti, Giuseppe Passarino, Francesco De Rango, Patrizia D'Aquila, Cristina Giuliani, Elena Marasco, Sebastiano Collino, Patrick Descombes, Paolo Garagnani, Claudio Franceschi

**Affiliations:** ^1^ Human Genetics, David Geffen School of Medicine, University of California Los Angeles, Los Angeles, CA 90095, USA; ^2^ Biostatistics, School of Public Health, University of California Los Angeles, Los Angeles, CA 90095, USA; ^3^ Department of Experimental, Diagnostic and Specialty Medicine, University of Bologna, 40126 Bologna, Italy; ^4^ Interdepartmental Center “L. Galvani”, University of Bologna, 40126 Bologna, Italy; ^5^ Personal Genomics S.r.l., 37134 Verona, Italy; ^6^ Istituto Auxologico Italiano IRCCS, Cusano Milanino, 20095 Milan, Italy; ^7^ Functional Genomics Center, Department of Biotechnology, University of Verona, 37134 Verona, Italy; ^8^ Geriatric Unit, Department of Medical Sciences and Community Health, University of Milan, 20122 Milan, Italy; ^9^ Geriatric Unit, Fondazione IRCCS Ca' Granda, Ospedale Maggiore Policlinico, 20122 Milan, Italy; ^10^ Department of Experimental and Clinical Biomedical Sciences, University of Florence, 50139 Florence, Italy; ^11^ Department of Cell Biology, University of Calabria, 87036 Rende, Italy; ^12^ Department of Biological, Geological and Environmental Sciences, Laboratory of Molecular Anthropology and Centre for Genome Biology, University of Bologna, 40126 Bologna, Italy; ^13^ Molecular Biomarkers, Nestlé Institute of Health Sciences SA, EPFL Innovation Park, 1015, Lausanne, Switzerland; ^14^ Functional Genomics, Nestlé Institute of Health Sciences SA, EPFL Innovation Park, 1015, Lausanne, Switzerland; ^15^ CRBA, Center for Applied Biomedical Research, St. Orsola-Malpighi University Hospital, 40138 Bologna, Italy; ^16^ CNR, Institute of Organic Synthesis and Photoreactivity (ISOF), 40129 Bologna, Italy; ^17^ IRCCS, Institute of Neurological Sciences of Bologna, 40139 Bologna, Italy

**Keywords:** semi-supercentenarians, semi-supercentenarians offspring, DNA methylation, epigenetic clock, biomarker of ageing

## Abstract

Given the dramatic increase in ageing populations, it is of great importance to understand the genetic and molecular determinants of healthy ageing and longevity. Semi-supercentenarians (subjects who reached an age of 105-109 years) arguably represent the gold standard of successful human ageing because they managed to avoid or postpone the onset of major age-related diseases. Relatively few studies have looked at epigenetic determinants of extreme longevity in humans. Here we test whether families with extreme longevity are epigenetically distinct from controls according to an epigenetic biomarker of ageing which is known as “epigenetic clock”. We analyze the DNA methylation levels of peripheral blood mononuclear cells (PBMCs) from Italian families constituted of 82 semi-supercentenarians (mean age: 105.6 ± 1.6 years), 63 semi-supercentenarians' offspring (mean age: 71.8 ± 7.8 years), and 47 age-matched controls (mean age: 69.8 ± 7.2 years). We demonstrate that the offspring of semi-supercentenarians have a lower epigenetic age than age-matched controls (age difference=5.1 years, p=0.00043) and that centenarians are younger (8.6 years) than expected based on their chronological age. By contrast, no significant difference could be observed for estimated blood cell counts (such as naïve or exhausted cytotoxic T cells or helper T cells). Future studies will be needed to replicate these findings in different populations and to extend them to other tissues. Overall, our results suggest that epigenetic processes might play a role in extreme longevity and healthy human ageing.

## INTRODUCTION

Ageing researchers and the general public have long been intrigued by centenarians because these subjects managed to avoid, postpone or overcome the major age-related diseases such as cancer [1], cardiovascular diseases [2], diabetes [3], osteoporotic fractures [4] and dementia [5, 6].

We find it useful to further distinguish centenarians from semi-supercentenarians (*i.e*. subjects that reach the age of 105 years, 105+) and supercentenarians (subjects that reach the age of 110 years, 110+) because subjects in these latter categories are extremely rare. As of January 1, 2015, in Italy 100+ are 19,095 out 60,795,612 living individuals, 105+, which constitute a subgroup of 100+, are 872 (1:69,720 living individuals) and 110+, which constitute an even smaller subgroup, are 27 (1:2,251,689 living individuals), according to the data base from the Italian National Institute of Statistics [7]. On the whole, 105+ and 110+ subjects have to be considered very rare cohorts of particular interest for the study of both the ageing phenotype and the healthy ageing determinants. This means that 105+ and 110+ are most informative for ageing research, even if it is not yet known whether 105+ reach the last decades of their life according to a molecular trajectory which progresses at a normal rate of change or whether the attainment of this remarkable age results from a slower molecular ageing rate.

A rich literature describes the relationship between blood-based markers and age [8–12] and many genetic studies were devoted to clarify whether exceptional longevity is a highly heritable trait [13–17]. More recently, a variety of “omics” studies have looked at gene expression [18–21], metagenomic [22] or lipidomic [23, 24] data. Different from genetic studies, functional genomic studies of centenarians face the challenge of identifying a proper control group, as shorter-lived controls from their birth cohort are no longer available. To address this challenge, we decided to compare the centenarians' offspring (CO) with the offspring of shorter-lived controls. CO are useful for finding suitable molecular markers and for estimating the trajectories of healthy ageing [25] because: i) longevity runs in families, which probably reflects shared genetic, epigenetic and environmental factors; ii) CO are on average 20-30 years younger than their centenarian parent, *i.e*. they are in their seventies or eighties, which is a critical age when the physiological decline and the onset of the major age-related diseases may occur; iii) it is feasible to recruit controls for CO that are age-matched and born from non-centenarian parents [25]. The comparison of CO to age matched controls has already been successfully applied to identify biochemical and metabolomics parameters related to exceptional longevity [24, 26–31] and to define survival scores [32, 33].

Many biomarkers of different origin have been used to disentangle the complexity underlying the ageing phenotype, *e.g*. inflammatory biomarkers [28–31], N-glycans [34–37] and telomere length. Telomere length is an attractive biomarker of ageing because a) telomere length shortening plays an essential role in the *in vitro* ageing of somatic cells and b) telomeres of different organs/cells are known to shorten with age [38–42]. While telomere erosion is clearly linked to ageing, a rich body of literature suggests that it is not the sole reason for *in vivo* ageing. For example, no significant association could be observed between telomere length and survival among the elderly and oldest old in Danish [43] and Japanese [28] populations.

Several recent studies propose biomarkers of ageing based DNA methylation levels [44–49]. DNA methylation levels give rise to powerful epigenetic bio-markers of ageing since chronological age (*i.e*. the calendar years that have passed since birth) has a profound effect on DNA methylation levels in most human tissues and cell types [50–59]. While previous epigenetic biomarkers of ageing apply to a single tissue, the recently developed “epigenetic clock” (based on 353 dinucleotide markers known as Cytosine phosphate Guanines or CpGs) applies to most human cell types, tissues, and organs [48]. Predicted age, referred to as “DNA methylation age” (DNAm age), correlates with chronological age in sorted cell types (CD4 T cells, monocytes, B cells, glial cells, neurons), tissues and organs including whole blood, brain, breast, kidney, liver, lung, saliva [48] and even prenatal brain samples [60]. The epigenetic clock is an attractive biomarker of ageing because a) it applies to most human tissues, b) its accurate measurement of chronological age is unprecedented [61], c) it possesses independent predictive value for all-cause mortality [62], d) it correlates with measures of cognitive and physical fitness in the elderly [63] and e) it has been found useful for detecting accelerated ageing effects due to obesity [64], Down syndrome [65] and HIV infection [66]. Furthermore, it demonstrates that the cerebellum ages more slowly than other brain regions [67].

Here, we analyze a novel peripheral blood mononuclear cells (PBMCs) methylation data set in an unprecedented Italian population of 105+, in their relative CO and in a cohort of healthy controls age- and sex-matched in respect of the CO group in order to test the hypothesis that these families age slowly according to the epigenetic clock.

## RESULTS

### Data set

We used the Illumina Infinium 450K array to generate DNA methylation data from PBMCs of 192 Italian subjects. We removed 8 samples (7 semi-supercentenarians and 1 control) from the analysis because they were potential outliers according to an unsupervised hierarchical clustering analysis based on the inter-array correlation. Our subsequent epigenetic clock analysis involved 3 distinct groups. The first group involved 75 subjects (mean age: 106 years, age range from 99 to 113 years) will be referred to as semi-supercentenarians (105+) although it included one subject aged 99. The second group, CO, involved 63 offspring from centenarians (mean age: 72 years, age range from 50 to 89 years). The third group involved 46 control subjects (mean age: 70 years, age range from 52 to 85 years), *i.e*. subjects who did not have a centenarian parent. The first group (semi-supercentenarians), the second (CO) and the third group (controls) contained 59, 25 and 37 females, respectively. By design, CO did not differ from controls in terms of gender (p=0.8) or chronological age (p=0.31).

### Accuracy of the epigenetic clock

DNAm age (also referred to as “epigenetic age”) was calculated using the DNA methylation levels of PBMCs applying a previously described method [48].

**Figure 1:**
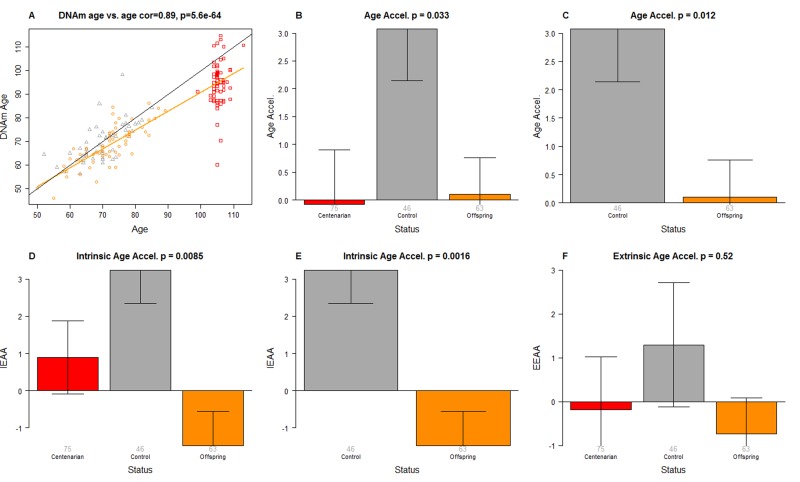
Epigenetic age analysis of PBMCs from centenarians and controls (**A**) Scatter plot relating the DNAm age of each PBMC sample (y-axis) versus chronological age (x-axis). Points are colored by status. The color of each dot corresponds to the status of each PBMC sample: red for centenarians, orange for offspring of centenarians, grey for controls. The black line corresponds to y=x. The orange line depicts the regression line based on the offspring of centenarians and centenarians (orange and red dots). The vertical distance to the orange line corresponds to the universal measure of age acceleration Age Accel. The bar plots depict group status (x-axis) versus (**B**, **C**) universal age acceleration, (**D**, **E**) intrinsic age acceleration, (**F**) extrinsic age acceleration. Each bar plot depicts the mean value, one standard error, and reports the p-value results from a non-parametric group comparison test (Kruskal Wallis test).

DNAm age was highly correlated with chronological age across all samples (correlation r=0.89, Figure 1A).

### Three measures of epigenetic age acceleration

In this article, we consider three measures of epigenetic age acceleration (as detailed in Methods). The first one, which will be referred to as universal measure of age acceleration (denoted Age Accel), applies to virtually all tissues and cell types (with the exception of sperm) [48]. The other two measures (referred to as intrinsic and extrinsic age acceleration, respectively) only apply to blood. The universal measure is defined as the difference between DNAm age value and the value predicted by the linear regression model in groups 1 and 2 (*i.e*. in semi-supercentenarians or their offspring). The term “universal” refers to the fact that this measure can be defined in a vast majority of tissues and cell types [48]. A positive value of the universal age acceleration measure indicates that DNA methylation age is higher than that predicted from the regression model for COs or semi-supercentenarians of the same age.

The measure of intrinsic epigenetic age acceleration (IEAA) measures “pure” epigenetic ageing effects in blood that are not confounded by differences in blood cell counts.

The measure of extrinsic epigenetic age acceleration (EEAA) aims to measure ageing in immune related components also relates to age-related changes in blood cell composition such as the decrease of naive CD8+ T cells and the increase in memory or exhausted CD8+ T cells [29, 68–70]. EEAA is defined on the basis of a weighted average of the epigenetic age measure from Hannum et al (2013) [47] and three blood cell types that are known to change with age: naive (CD45RA+CCR7+) cytotoxic T cells, exhausted (CD28-CD45RA-) cytotoxic T cells and plasma B cells. By definition, EEAA has a positive correlation with the amount of exhausted CD8 T cells and plasma blast cells and a negative correlation with the amount of naive CD8+ T cells. Blood cell counts were estimated based on DNA methylation data as described in Methods (section “Estimating blood cell counts based on DNA methylation levels”).

The three different measures of epigenetic age acceleration are not independent of each other. The universal measure Age Accel has a moderately high correlation with IEAA (r=0.65, p=1.8×10^−23^) and with EEAA (r=0.71, p=1.6×10^−29^). But IEAA has only a weak correlation with EEAA (r=0.19, p=0.01). By construction, our three measures of epigenetic age acceleration are uncorrelated (r=0) with chronological age at the time of blood draw.

### Offspring of semi-supercentenarians have a slow intrinsic ageing rate

We find that PBMCs of the offspring of 105+ age more slowly than that of age matched controls according to a) the Age Accel measure (Kruskal Wallis test p=0.012, Figure 1C) and b) the intrinsic measure of age acceleration (p=0.0016 Figure 1E). According to a multivariate model analysis in non-centenarians (Table 1), CO are 5.1 years younger (p=0.00051) than age matched controls even after adjusting for sex and estimated blood cell counts.

**Table 1:** Multivariate model of DNAm age in non-centenarians

Covariate	Coefficient	Std. Error	T statistic	P-value
Offspring	−3.804	1.043	−3.647	0.00043
Age	0.743	0.071	10.442	< 2×10^−16^
Sex (female)	0.174	1.065	0.163	0.87
Naïve CD8+T cell	−0.007	0.011	−0.617	0.54
Exhausted CD8+ T cell	−0.044	0.159	−0.274	0.79
Plasma Blast cell	−7.62	3.706	−2.055	0.042
Helper T cell (CD4)	−28.3	8.728	−3.247	0.0016
Natural Killer cell	12.5	7.962	1.572	0.13
Monocyte	−9.2	9.313	−0.988	0.33

Coefficients and p-values from regressing DNAm age on offspring status, age, sex, and various blood cell counts in non-centenarian subjects (subjects younger than 90). The model explained 67% of the variance. The offspring of centenarians are 5.1 years (=3.804/0.743) younger than age matched controls.

### Semi-supercentenarians appear to age more slowly than expected

Interestingly, the DNAm age of 105+ is systematically lower than their chronological age as can be seen from the fact that the red dots lie beneath the black line in Figure 1A. The DNAm age of 105+ differs significantly from that of controls (p=0.028) but not from that of the CO (p=0.29) according to a multivariate model analysis (Table 2) that adjusted for chronological age, sex, and estimated blood cell counts. According to this model, 105+ are on average 8.6 years younger than expected based on chronological age. The systematic underestimate of age in centenarians has also been observed in most other tissues from centenarians [67].

**Table 2:** Multivariate model of DNA methylation age in all subjects

Covariate	Coefficient	Std. Error	T statistic	P-value
StatusControlvsCentenarian	6.583	2.98	2.208	0.029
StatusOffspringvsCentenarian	3.03	2.84	1.069	0.29
Age	0.765	0.080	9.578	< 2×10^−16^
Sex (female)	−1.025	1.02	−1.005	0.32
Naïve CD8+T cell	−0.014	0.010	−1.444	0.15
Exhausted CD8+ T cell	−0.118	0.142	−0.832	0.41
Plasma Blast cell	−6.046	2.83	−2.135	0.034
Helper T cell (CD4)	−38.069	7.53	−5.054	1.1×10^−6^
Natural Killer cell	−1.926	7.25	−0.265	0.79
Monocyte	−14.782	8.00	−1.848	0.066

Coefficients and p-values from regressing DNAm age on offspring status, age, sex, and various blood cell counts in non-centenarian subjects (subjects younger than 90). The model explained 85% of the variance. Centenarians are 8.6 years (=6.583/0.765) younger than expected based on chronological age.

While our results in the CO suggests that the age difference of 8.6 years reflects a lower epigenetic ageing rate in 105+, we cannot rule out that confounders play a role as well (due to the lack of suitable controls for centenarians).

### Extrinsic age acceleration and blood cell counts are not significant

**Figure 2:**
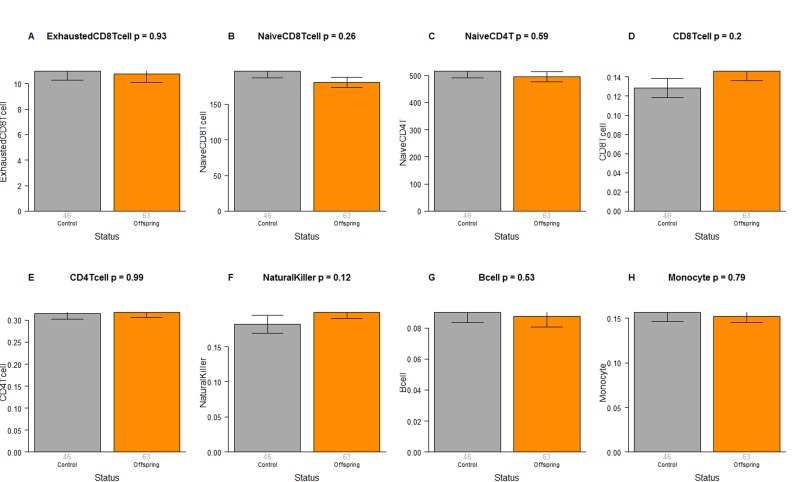
Blood cell counts in offspring of semi-supercentenarians versus age matched controls Group status (offspring of semi-supercentenarian or control) versus estimated abundance of (**A**) exhausted cytotoxic T cells, (**B**) naïve cytotoxic T cells, (**C**) naïve helper T cells, (**D**) cytotoxic T cells, (**E**) helper T cells, (**F**) natural killer cells, (**G**) B cells, (**H**) monocytes. Each bar plot reports the mean value and one standard error. The p-value results from a non-parametric group comparison test (Kruskal Wallis). The abundance measures of blood cell counts were estimated based on DNA methylation levels using the epigenetic clock software. Roughly speaking, the y-axis of (**A**) reports a percentage while that for (**B**, **C**) corresponds to counts but it is best to interpret the y-axis in (**A**-**C**) as ordinal abundance measure. The y-axis in (**D-H**) reports estimated proportions based on the Houseman method [78].

**Figure 3:**
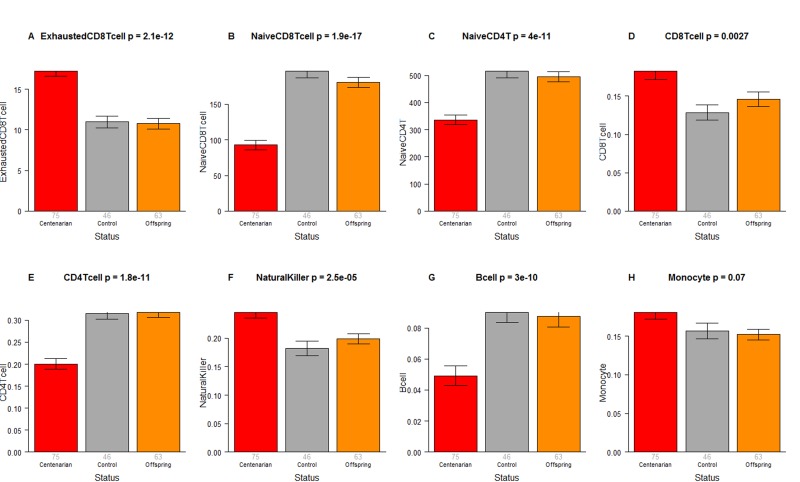
Blood cell counts across three groups Group status (semi-supercentenarian, offspring of semi-supercentenarian, or control) versus estimated abundance of (**A**) exhausted cytotoxic T cells, (**B**) naïve cytotoxic T cells, (**C**) naïve helper T cells, (**D**) cytotoxic T cells, (**E**) helper T cells, (**F**) natural killer cells, (**G**) B cells, (**H**) monocytes. Each bar plot reports the mean value and one standard error. The p-value results from a non-parametric group comparison test (Kruskal Wallis). Roughly speaking, the y-axis of (**A**) reports a percentage while that for (**B**, **C**) corresponds to counts but it is best to interpret the y-axis in (**A**-**C**) as ordinal abundance measure. The y-axis in (**D**-**H**) reports estimated proportions based on the Houseman method [78].

The CO do not differ from age matched controls in terms of the extrinsic measure of age acceleration (Figure 1F) or in terms of estimated blood cell counts (Figure 2) but future studies with large samples should revisit these analyses. When comparing 105+ to younger subjects (CO and controls), we find that 105+ contain more exhausted cytotoxic T cells (Figure 3A), fewer naïve cytotoxic T cells (Figure 3B), fewer naïve helper T cells (Figure 3C), more cytotoxic T cells (Figure 3D), fewer helper T cells (Figure 3E), more natural killer cells (Figure 3F), and fewer B cells (Figure 3G). These findings are congruent with those from many previous studies of age related changes in blood cell composition (e.g. [10, 29, 68–72]).

## DISCUSSION

This article leverages three epigenetic biomarkers of ageing to characterize semi-supercentenarians and their offspring.

To the best of our knowledge, this is the first study that demonstrates that the offspring of semi-supercentenarians have a lower intrinsic epigenetic ageing rate in PBMCs. A similar trend was reported by Gentilini [73] that, by analyzing whole-genome methylation data from PBMCs in a smaller cohort (including 21 female centenarians, their 21 female offspring, 21 offspring of both non-long-lived parents and 21 young women), observed an age-related decrease in global DNA methylation and a delay of this process in centenarians' offspring. In the present study, the reported p-values (p=0.00051 in Table 1, p=0.012 in Figure 1C) remain significant even after adjusting for multiple comparisons since our study only involved three major hypothesis tests corresponding to the three related measures of epigenetic age acceleration (*i.e*. Age Accel, IEAA, EEAA). This remarkable finding is mirrored by the result that semi-supercentenarians appear to be younger (8.6 years, Table 2) than expected based on chronological age. Future studies will be needed to investigate how an epigenetic trajectory of healthy ageing relates to that of clinical measures of physiological or pathological ageing.

Strengths of this study include a) access to PBMCs from a unique collection of semi-supercentenarians and their offspring; b) careful matching between the offspring of semi-supercentenarians and unrelated control offspring from non-centenarians; c) state of the art epigenetic biomarkers of ageing. The following limitations need also to be acknowledged. First, our results should be replicated in other populations (ideally involving semi-supercentenarians and their offspring) who differ from our Italian cohort in terms of genetic background, lifestyle and cultural habits. Further, it will be of great interest to extend this kind of epigenetic clock analysis to other accessible fluids and tissues such as buccal epithelium, saliva, skin, adipose, muscle. It is beyond the scope of this article to carry out an epigenome wide association studies (EWAS) based on 486k individual CpGs on the Illumina array. The number of semi-supercentenarians included in this study is relatively small but these subjects are very rare, *i.e*. about one in 69,720 Italian living individuals and 6 out of the top 50 oldest living people are Italian (https://en.wikipedia.org/wiki/List_of_oldest_living_people).

The epigenetic clock and related methylation-based biomarkers of ageing are arguably the most accurate measures of the wider process of epigenetic remodeling that occurs in different cell types, tissues and organs during human ageing [46, 48, 74, 75]. Previous studies have shown that epigenetic age relates to cognitive status, physical fitness, and all-cause of mortality in aged populations [62, 63, 79–81]. The current study adds to an increasing body of evidence that suggests that the epigenetic age of PBMCs relates to biological age or physiological age but other complementary biomarkers of ageing undoubtedly play a crucial role. It is highly unlikely that a single blood-based biomarker of ageing (such as epigenetic age) captures all aspects of physiological age.

Overall, our results suggest that the offspring of semi-supercentenarians are informative when it comes to detecting epigenetic determinants of healthy ageing. By understanding why the offspring of centenarians are protected against epigenetic ageing, we might be able to learn how to extend the benefits of successful ageing to the general population.

## MATERIALS AND METHODS

### Description of the dataset

The subjects were recruited in three Italian centers (Bologna , Milan and University of Calabria at Rende). The study was approved by the local Ethical Committee (S. Orsola Hospital - University of Bologna; Prot. n. 2006061707, amendment 08/11/2011; Fondazione IRCCS Cà Granda Ospedale Maggiore Policlinico, Prot. n. 2035, amendment 30/11/2011; University of Calabria 9/9/2004 amendment on 24/11/2011). A written informed consent form was obtained from all participants.

This novel dataset (measured on the Illumina 450K array) includes 192 subjects: 82 semi-supercentenarians (33 from Bologna, 29 from Milan and 20 from Calabria), 63 offspring of semi-supercentenarians (22 from Bologna, 28 from Milan and 13 from Calabria) and 47 control subjects whose parents were not centenarians (16 from Bologna, 17 from Milan and 14 from Calabria).

### DNA extraction and bisulphite treatment of DNA

Extraction of genomic DNA from PBMCs was performed using the AllPrep DNA/RNA/protein kit (QIAGEN, Hilden, Germany). Sodium bisulphite conversion for Infinium HumanMethylation450 BeadChip was performed using the EZ-DNA Methylation-Gold Kit and the EZ-96 DNA Methylation Kit respectively Genome-wide DNA methylation was analyzed using the Infinium HumanMethylation450 BeadChip (Illumina, San Diego, CA) following manufacturer's instructions. Arrays were scanned by HiScan (Illumina). GenomeStudio (Illumina) was used to perform background subtraction.

### DNA methylation age and epigenetic clock

The epigenetic clock software implements a data normalization step that repurposes the BMIQ normalization method from Teschendorff [76] so that it automatically references each sample to a gold standard based on type II probes as detailed in Additional file 2 from [48]. All of the described epigenetic measures of ageing and age acceleration are implemented in our freely available software [48]. The epigenetic clock is defined as a prediction method of age based on the DNA methylation levels of 353 CpGs. Predicted age, referred to as DNAm age, correlates with chronological age in sorted cell types (CD4 T cells, monocytes, B cells, glial cells, neurons), tissues and organs, including: whole blood, brain, breast, kidney, liver, lung, saliva [48]. Mathematical details and software tutorials for the epigenetic clock can be found in the Additional files of [48]. An online age calculator can be found at our webpage (https://dnamage.genetics.ucla.edu).

### Measures of epigenetic age acceleration

The name of our universal measure of age acceleration (Age Accel) reflects that it applies to virtually all sources of human DNA (with the exception of sperm). Here we defined it as follows. First, we regressed DNAm age on chronological age in semi-supercentenarians and their offspring. Next, we used the resulting model to predict the age subject. Next the universal measure was defined as the difference between the observed measure of DNAm age and the predicted value. Thus, a high positive value for Age Accel indicates that the observed DNAm age is higher than that predicted based on semi-supercentenarians and their offspring. Age Accel has a relatively weak correlation with blood cell counts [66] but it still relates to estimated blood cell counts as can be seen from Table 1. To subtract out the effect of blood cell counts, we find it useful to define a measure of intrinsic epigenetic age acceleration (IEAA) that measures “pure” epigenetic ageing effects that are not confounded by differences in blood cell counts. It is defined as the residual resulting from a multivariate regression model of DNAm age on chronological age and various blood immune cell counts (naive CD8 T cells, exhausted CD8 T cells, plasma B cells, CD4 T cells, natural killer cells and monocytes).

The measure of extrinsic epigenetic age acceleration (EEAA) aims to measure epigenetic ageing in immune related components. EEAA is defined using the following three steps. First, we calculated the epigenetic age measure from Hannum et al (2013) [47], which is weakly correlated with certain blood cell types [62]. Second, we increased the contribution of blood cell types to the age estimate by forming a weighted average of the Hannum's estimate with 3 cell types that are known to change with age: naive (CD45RA+CCR7+) cytotoxic T cells, exhausted (CD28-CD45RA-) cytotoxic T cells, and plasma B cells using the approach of [77]. The resulting measure of blood age is referred to as BioAge4 in our epigenetic clock software. Third, we defined a measure of age acceleration (EEAA) as the residual resulting from a univariate model regressing BioAge4 on chronological age. By definition, our measure of EEAA has a positive correlation with the amount of exhausted CD8 T cells and plasma blast cells and a negative correlation with the amount of naive CD8+ T cells. Blood cell counts were estimated based on DNA methylation data as described in the section entitled “Estimating blood cell counts based on DNA methylation levels”. By construction, EEAA tracks both age related changes in blood cell composition and intrinsic epigenetic changes. By definition, none of our three measures of epigenetic age acceleration are correlated with the chronological age.

### Estimating blood cell counts based on DNA methylation levels

We estimate blood cell proportions using two different software tools. Houseman's estimation method [78], which is based on DNA methylation signatures from purified leukocyte samples, was used to estimate the proportions of cytotoxic (CD8+) T cells, helper (CD4+) T, natural killer B cells. The advanced analysis option of the epigenetic clock software [48] was used to estimate the percentage of exhausted CD8+ T cells (defined as CD28-CD45RA-) and the number (count) of naïve CD8+ T cells (defined as (CD45RA+CCR7+).
